# Global migration governance from below in times of COVID-19 and “Zoomification”: civil society in „invited “ and „invented “ spaces

**DOI:** 10.1186/s40878-021-00275-9

**Published:** 2022-01-06

**Authors:** Stefan Rother

**Affiliations:** University of Freiburg, Rempartstr. 15, 79085 Freiburg, Germany

**Keywords:** COVID-19, Global migration governance, Civil society, Human rights, Global compact for migration, United Nations, Social movement

## Abstract

The global pandemic has resulted in ad hoc unilateral policies on migration, mobility and border management while at the same time emphasizing the need for global cooperation. For global governance in this field to be effective, it needs to include stakeholders beyond states and international institutions. The Global Compact for safe, orderly and regular Migration (GCM) highlights the role of those groups directly affected by global policies, i.e. migrants and their organisations. The goal of this paper is to analyse the role of civil society in global migration governance in times of COVID-19. It employs a comparative approach between “invented” and “invited” spaces. “Invited spaces” in this context refer to spaces created by international organisations such as the United Nations Network on Migration’s “Stakeholder Listening Sessions” on COVID-19 and the resulting statements. “Invented Spaces” refer to self-organized spaces by civil society actors. The paper will compare these spaces regarding their openness, the central issues and calls for specific policy measures, the stakeholders involved and the strategies they employ. I argue that the pandemic has strengthened the “input” dimension for migrant civil society in global governance. This relates to the structure/format as well as to the content of the participation. “Zoomification” has opened up access to “invited” spaces while pushing forward the creation and scope of “invented” spaces”. There are indicators that the pandemic has also influenced parts of the output dimension, although it is too early to assess whether this will have a lasting effect on policies on the ground.

## Introduction

This article analyses the participation of civil society in deliberations on the global governance of migration. I argue that the pandemic has influenced these deliberations in two major regards: First, by creating new and intensifying existing challenges for a rights-based approach to migration; and second, by thoroughly affecting the access to and forms of deliberations within spaces of global migration governance. The article compares the political opportunity structures in formal spaces to which civil society is “invited” and those created by civil society itself (“invented”) and discusses the effect of the COVID-19 pandemic on these processes. This relates particularly to the “zoomification” of meetings: has moving the deliberations into the virtual space primarily been a hindrance for meaningful multi-stakeholder participation or does it provide a new window of opportunity as well? And how has COVID-19 affected the boundaries between formal spaces to which civil society is “invited” and those created by civil society itself (“invented”)—are these reinforced or have they been blurred, i.e. have issues managed to “travel” between these spaces? This comparison is relevant in two regards: format and content.

Obviously, this is very much an ongoing process and looking at it from not much more than one year into a crisis mode does not yet allow for a strong conclusion. Certain trends can be observed, though, and based on them, I propose the following argument: 1. The pandemic has strengthened the “input” dimension for migrant civil society in global governance. This relates to the structure/format as well as to the content of the participation. Format refers to the way these meetings are structured, how access is granted, which political opportunity structures they provide. This can have an effect on the content—i.e. what is discussed and, as important, what is or cannot be discussed “Zoomification” has opened up access to “invited” spaces while pushing forward the creation and scope of “invented” spaces”. And the experience of the pandemic has opened up space for discussions on topics which so far have been considered as “taboos” by many states, namely regularization and access to public services independent of status. 2. There are indicators that the pandemic has also influenced the first stage of the output component—i.e. issues proposed by migrant civil society have found their way into statements and reports produced by the UN system or influenced the agendas of government meetings. It is too early, though, to assess if this will have a lasting effect on the second stage of the output, i.e. concrete policy measures, legislation etc. that affect the situation of migrants on the ground.

The research follows up on twelve years of participant observation in such spaces, including the Global Forum on Migration and Development (GFMD), the Intergovernmental Conference on the Global Compact for Migration, the People’s Global Action on Migration, Development and Human Rights (PGA), the World Social Forum on Migration (WSFM) and many more. As a result of the pandemic, physical meetings have been moved to fully virtual spaces, though. To explore what this means for civil society engagement in global migration governance, I have attended more than 40 web meetings and interactive webinars that addressed regional and global migration governance issues up until the online GFMD chaired by the United Arab Emirates (UAE) in January 2021. While some of those were streamed live on openly accessible channels such as YouTube and/or were uploaded afterwards, others were conducted under Chatham House rules.^1^ I also conducted several informal exchanges with stakeholders, for which the same rules apply. In addition, I conducted two in-depth-interviews with major organisers of “invited” and “invented” spaces via Zoom, who agreed to be quoted in person.

In order to properly assess the impact of COVID-19, I will start with a brief overview on the state of the global governance of migration before the pandemic hit. I will then provide a brief theoretical background on the role of migrant civil society in global migration governance and the concept of “invited” and “invented” spaces. This will be followed by sections analysing the participation in both these spaces, the stakeholders involved and the emerging central advocacy and policy issues. In the conclusion, I will reflect upon whether the “virtualisation” of participatory spaces can contribute to the democratisation of the deliberations on global migration governance.

## The state of global migration governance

Before the pandemic, several advancements had been made in global migration governance, with the adoption of the United Nations Global Compact for safe, orderly and regular migration (GCM) in 2018 being the most prominent achievement. The document was seen as a major milestone, providing through its 23 objectives a “360 degree vision” on migration. It was not without its critics, though. An increasing populist backlash against the compact could be observed in the months leading up to the adoption and some states backed out citing concerns over their sovereignty—an argument that did not hold up under closer scrutiny, since the GCM is explicitly a non-binding document. Vice versa, this aspect was criticised by several civil society representatives, who had been calling for a legally binding instrument similar to the 1990 International Convention on the Protection of the Rights of All Migrant Workers and Members of Their Families (ICRMW) (Schierup et al., 2019). In response, proponents of the GCM pointed towards the low number of ratifications this core UN human rights treaty has managed to gather—after 30 years they amount to only 56, none of them from a major country of destination. In contrast, the open nature of the compact was promoted as an advantage since it would make it easier to get more states on board.

The non-binding nature of the GCM notwithstanding, it provides for a review mechanism; the first International Migration Review Forum is scheduled for May 2022 and in late 2020 the regional reviews for the first steps of compact implementation had been started.

Finally, the compact was introduced not as an isolated document but rather as a “living framework”. The process of getting there was seen as fairly transparent and inclusive with many stakeholders being consulted; in particularly, migrants right networks and their umbrella organisations had been able to bring their advocacy into the deliberations of the compact and influence the final document with regards to aspects such as family reunification as well as the rights of undocumented migrant workers and their access to services regardless of migration status (Rother & Steinhilper, 2019).

Parallel to the compact deliberations, a reshaping of the global migration governance landscape within and around the UN was taking place. The International Organisation for Migration (IOM) that had so far been an independent organisation reporting to its own membership, moved closer to the UN system as a “related organisation”. It also helped establish the newly formed United Nations Network on Migration (from here on “the Network”) and staffed its secretariat with the goal of better cooperation among the UN entities dealing with migration (a task in which the predecessor of the Network, the Global Migration Group, had by all accounts not succeeded).

In sum, the past few years had seen an increasing dynamic and willingness to address migration as an issue that requires multilateral cooperation. There seemed to be an overall consensus that the compact would be at the centre of global deliberations on migration for years to come. Accordingly, during the 12^th^ summit of the Global Forum on Migration and Development (GFMD) in January 2020 in Quito, Ecuador, the agenda was dominated by the sharing of good practices in implementing the GCM and by expanding the multi-level scope of global migration governance through the inclusion of further stakeholders such as the Mayoral Forum on Human Mobility, Migration and Development (Mayoral Forum).

However, only a few weeks later, when the serious nature of the COVID-19 pandemic started to become widely apparent, multilateral cooperation on issues of migration and mobility was nowhere to be seen. Instead, borders were being closed by unilateral decisions, migrants were deported and/or became stranded and even non-migrants experienced a significant limitation of their mobility. And while the GCM could have provided guidance on issues relevant during the pandemic such as consular protection and assistance, support for vulnerable migrants, access to basic services, remittances transfer and financial inclusion of migrants (Newland, 2020), it was hardly ever referred to in national policymaking. Furthermore, the unilateral actions of national governments who often did not consult other states and even less so migrant civil society, stood in stark contrast to the “whole-of-society approach” laid out in the “vision and guiding principles” of the GCM: address migration in all its dimensions by including migrants, diasporas, local communities, civil society, academia, the private sector, parliamentarians, trade unions, national human rights institutions, the media and other relevant stakeholders in migration governance” (United Nations General Assembly, 2019: 6).

## Migrant civil society in “invited” and “invented” spaces of participation

While the term “civil society” is often used as an umbrella term for non-state actors besides the private sector, it obviously refers to a very heterogenous group. The same holds true for migrant civil society (Rother, 2019a). The term encompasses support organisations for migrants by “concerned citizens” or faith-based groups as well as a multitude of forms of migrant self-organizing ranging from service-oriented to explicitly political organisations, acting at the grassroots- as well as (trans)national and regional level of politics. As diverse as these actors might be, over the years many of them have formed alliances and “networks of networks (inter-movement building)” (Piper, 2015: 795) reaching up to the global level. The main focus of these umbrella organisations is a rights-based approach to migration; stressing that human rights do not end at borders, advocating for protection, assistance and justice in particular for migrant workers and their families. It can be argued that bringing migrants’ voices, experiences and demands into deliberations can contribute to a—low-level—form of democratizing global governance and international institutions by providing migrants with agency rather than being mere objects of governance (Rother, 2013).

The network-building of migrant civil society on the global level started out as being rather reactive: when migration began to slowly enter the arena of global politics at the turn of the millennium, space for civil society participation was very limited and there was a need to select representatives who could fulfil the accreditation criteria. Hence, the NGO Committee on Migration (CoM) was constituted in Consultative Status with the United Nations Economic and Social Council (ECOSOC) as a response to the first global meeting specifically dedicated to migration—the UN High-Level Dialogue on Migration and Development (UN-HLD) in 2006 (Rother, 2020). As a follow-up, the GFMD was established and the International Catholic Commission on Migration (ICMC) played an increasingly significant role, first as a representative, then from 2011 onwards as organizer for the dedicated Civil Society Days within the forum. And when in 2016 the UN High-Level Meeting (HLM) that would lead to the establishment of the compacts on migrants and refugees was announced, these two actors joined forces with other stakeholders and established the Civil Society Action Committee (from here on “Action Committee”) with the goal to represent migrant’s voices in the deliberations on and implementation of the GCM.

Over the course of one and a half decades of organizing, migrant civil society representatives had come to the conclusion that it would not be sufficient to just show up in the limited spaces provided for them by governments and global institutions. Rather, they had to create their own independent spaces for broader, more open and inclusive exchanges on migration policies. To analyse these strategies, the concepts of “invited” and “invented” spaces for civil society participation can be useful.

*Invited spaces* can either refer to existing spaces that are opening up for civil society participation or new spaces that are specifically created for such participation. In both cases, civil society actors are rather guests than hosts (although they might be involved in the organisation and selection process of participants). *Invented spaces*, on the other hand, are characterized by a higher degree of civil society autonomy on issues such as the agenda, mode of operation and participant selection. The two spaces can thus differ in format and content and the latter can be related to the former, i.e. in more open and autonomous spaces a wider range of issues, including controversial ones, can be discussed.

The concept of *invited spaces* was gathering increasing attention in the early 2000s (Brock et al. 2001). In an early definition, Andrea Cornwall described them as “those into which people (as users, citizens or beneficiaries) are invited to participate by various kinds of authorities, be they government, supranational agencies or non-governmental organisations” (Cornwall 2002). These spaces are framed by those who create them, and are reflections of power relations, in which certain actors can be silenced; but they can also become “spaces of possibility” where citizens can take on a more active role (Cornwall & Coelho, 2007: 11). This approach shares similarities to the concept of created spaces (Gerard, 2014) where “collective action is mobilized in a way that does not require the sanction of governmental authorities” (Jayasuriya & Rodan, 2007: 786).

The early works on “invited spaces” often put a strong focus on direct citizen participation, i.e. on the local level, in their empirical analysis. However, it is also made clear that the spaces can be positioned at or stretch over multiple levels; in his “power cube” model, Gaventa identifies the local, national and global level (bypassing the transnational and regional level, particularly relevant for migration) and characterizes the spaces as either “closed”, “invited” or “claimed/created” (Gaventa, 2006: 25). The latter spaces are created by less powerful actors who share common concerns and identities and can be seen as counter-hegemonic (Soja, 1996)—either through their very existence or explicitly by design.

Miraftab refers to such spaces *as invented spaces*, where grassroots movements are challenging the *status quo* (Miraftab, 2004). She points out, though, that invited and invented “spaces of practicing citizenship are not mutually exclusive” (Miraftab, 2004: 3). It might in fact be a strategic decision to use every space available and try to connect them; major migrants rights network characterize this approach as an “inside-outside strategy” (Rother, 2009).

Ålund and Schierup have applied the invited and invented distinction to civic activism in the global governance of migration, where independent spaces are created by civil society not just as an addendum but as an alternative in order to avoid or counter co-optation into dominant discourses and power relations (Ålund & Schierup, 2018).

The primary focus of these studies is on physical spaces of participation. A rare exception is Norbert Kerstings work on “Online participation: from ‘invited’ to ‘invented’ spaces”” (Kersting, 2013). His assessment is rather critical, though; much of the participation he analyses falls under the category of “demonstrative democracy”, focusing on symbolic participation and expressivity. Kersting points towards “shit storms” and quickly escalating online discussions. He argues that in more formal online deliberations, participants might not be qualified and the communication is open for misuse and manipulation; and even when “the quality is enhanced with authentication, the quality of discourse is still miserable” (Kersting, 2013: 275). These are observations probably everyone who has ever been online can relate to and many of them have rather worsened in the time since Kerstings article was originally published. On the other hand, technology has advanced in these years and enabled even users with standard equipment to participate in more personal video-meetings. This development has made possible the “Zoomification”^2^ of global migration governance deliberations in the times of COVID-19. In the next sections, I will discuss and compare invited and invented spaces with regards to their format, the stakeholders involved and the content, i.e. the issues discussed.

## Invited spaces: the UN network on migration

As mentioned above, 2020 promised to be an intense year for deliberations on global migration governance. During the GFMD in Quito, Ecuador, the incoming chair of the forum, the UAE announced an ambitious work plan. This included six regional consultations leading up to the 13^th^ summit of the forum in Dubai in early 2021. Delegates were also surprised to learn about a new approach to this summit: rather than having separate Government Meetings and Civil Society Days, connected by a “common space” dedicated to mutual exchange, all five days of the summit were to be opened up to all stakeholders. While civil society had managed to expand its presence in the invited spaces of the GFMD process over the past years, this new approach could still be considered a major step towards civil society inclusion. At the same time, the regional review processes for the GCM were scheduled for 2020, with Jonathan Prentice, Head of Secretariat of the United Nations Network on Migration, admitting in a civil society meeting in Quito that there was confusion regarding “as to how these might take place and to what purpose” (Rother, 2020: 16).

The migration network itself was still in the process of establishing its structures and defining its role. Besides bringing together 38 entities in the UN system and supporting states in addressing their migration priorities, the mandate of the network is to “prioritize the rights and wellbeing of migrants and their communities of destination, origin, and transit”.^3^ To this regard, the network also engages with external partners and at the end of 2019, a seasoned migrant’s rights activist, Monami Maulik, was hired as Civil Society Liaison Officer.

The original work plan for 2020 focused on ground level implementation of the GCM. Several working groups had been set up: three core working groups on issues such as knowledge exchange and implementation and six thematic working groups on issues ranging from Bilateral Labour Migration Agreements to Returns and Reintegration. Their input was expected to lead to pilot projects.

When the pandemic became a global issue, though, the workplan had to stop since all the involved agencies, stakeholders and civil society organisations went into crises mode. This led to a period of reflection and strategizing within the network and Maulik pitched the idea of introducing listening sessions as a new format strengthening the input dimension of civil society in global migration governance: “When there is a crises moment, we should first hear from the people on the ground, the frontline of the crises. Because in such a time of crises, you do not have time to gain appropriate data and information, everything is happening as it is happening, and you have to listen to the people who are at the border, who are in the camp” (Interview Monami Maulik (MM), October 16, 2020).

This approach of creating new “invited spaces” was taken up by the network and a first session was held on April 1, 2020, titled “Listening Session about the ground-level impacts of COVID-19 on migrant communities”.^4^ In the invitation email the session was introduced as “a platform to exchange stories and anecdotal evidence, emerging trends and important response efforts underway from stakeholders and governments”. It was stated that the purpose of the sessions would reach beyond listening and learning—“these platforms for open exchange will help us collectively focus on the key issues facing migrants across country contexts and inform where the Network’s priorities should be. The Network seeks to add critical value in ensuring that migrants are included in immediate response and long-term measures impacting their health, economic and social well-being and human rights. Importantly, we also seek to highlight the critical contributions of migrants as frontline workers providing care, transport, health, food production and other services.” (Email March 30, 2020).

This statement is quite remarkable in that it ascribes agency to migrants and their organisations on three levels: first, by recognizing their efforts on the ground, second, as experts whose input could influence in which direction the Network would reprioritize its work, and third, as partners in responding to the challenges of COVID-19. It thus promises to include migrant civil society in the input as well as output dimension of global migration governance. The invitation had been preceded by a similarly inclusive statement of the Network, issued on March 20: “COVID-19 Does Not Discriminate; Nor Should Our Response”.^5^ Here, the Network “urges that all—including migrants regardless of migratory status—are included in efforts to mitigate and roll back this illness’s impact”. Several specific policy recommendations are made, including access to health services regardless of status, rights to adequate living standards, fighting xenophobia, upholding human rights and the right to seek asylum in times of tightened borders, labour rights for migrant workers and, carefully worded, a call for alternatives to immigrant detention centres.

The GCM is not explicitly mentioned in the document—somewhat surprisingly, considering that this statement touches upon several objectives of the compact, that are as essential as they were controversial in the negotiation process of the GCM. While for some states these issues were seen as an obstacle in getting behind the compact unless they were toned down, migrants rights organisations had hoped for stronger wording such as an explicit call for “firewalls” to be erected between access to public services and immigration enforcement authorities (Crépeau & Hastie, 2015). From a migrant’s right perspective, this is also a notable step forward from the language that dominated discourses in the early days of global migration governance; it took some time until rights were explicitly mentioned in the GFMD process and the rhetoric of IOM, which still considers itself to be a “non-normative organisation”, had so far rather called for “protection” and promoted “well-managed migration policies”. The shift towards a stronger rights-based language and clearer acknowledgement of migrants as subjects of migration governance could thus be seen as the result of “socialization” through years of engagement and deliberations (Rother, 2019b).

The two listening sessions provided space for a wide range of inputs that highlighted the truly global nature of the pandemic. To list just a small selection of pressing issues that came up; harassment and limited to no access to health services for refugees and asylum seekers in Australia, Pakistan and at the US-Mexico border; no safety nets for migrant street vendors in New York; ongoing deportations in Kuwait; no payment and food scarcity for migrant workers in Malaysia; widespread attacks against migrants of Chinese descendance; criminalization and scapegoating of migrant sex workers.

However, these sessions were not only about grievances– good practices were shared as well: the Bulgarian Red Cross providing food packages, cash assistance and hygiene items to undocumented migrants; the Spanish Ombudsman issuing a recommendation to release migrants from detention centres and finding them a safe place to stay, doing so in coordination with different administrations, since no repatriation operations were taking place; Portugal granting undocumented migrants and asylum seekers residence rights in order to mitigate their COVID exposure. This can also be seen as a framing strategy—states might more easily respond to specific policy measures already taken by other states than to more principled interventions by non-state actors.

Since the sessions were considered an important tool in times of heightened uncertainty, and the information gathered was deemed to be highly relevant, the network decided to follow those up with several more sessions with a thematic focus. Furthermore, a “Voices from the Ground” platform was launched on the Network’s COVID-19 web page that brought together statements, snapshots, blogpost and call for actions by non-state stakeholders.^6^ The selection of the topics of the follow-up listening sessions was to be ”demand-driven” and thus reflects the issues considered particularly pressing during the pandemic: “Safe and Inclusive Access to Services” (May 7), “Alternatives to Detention” (May 20), “Regular Pathways” (June 9), “Gender-specific Implications of the COVID-19 Pandemic on Migrants” (June 11), “Addressing the vulnerabilities of migrant workers in agriculture during and after the COVID-19 pandemic” (July 2) and “COVID-19 and Trafficking in Persons” (July 9).

Besides civil society, representatives of various UN agencies, member states and several other stakeholders participated. Monami Maulik stressed that these sessions were not just held for the sake of listening but would “actually shape how the UN system and the UN network is going to re-strategize, its direction on the GCM and implementation”. Specifically, the input would “feed directly into the working groups creating policy briefs on each of these topics for the member states” and thus contribute to the first stage of the output dimension. Migrant civil society could use this “invited space” to shape the input for a more restricted space, one primarily established for member states.

The roadmap was to conduct the listening sessions, take up examples and recommendations from hundreds of actors attending, develop policy briefs by the working groups and launch these with webinars so the member states could hear the policy recommendations. One such policy brief was published on June 11 under the title “Enhancing Access to Services for Migrants in the Context of COVID-19 Preparedness, Prevention, and Response and Beyond”.^7^ It was developed by the thematic Working Group on Access to Services under the co-leadership of WHO and UN-Habitat with support and contributions from various member institutions of the network as civil society organisations such as the Platform for International Cooperation on Undocumented Migrants (PICUM), a network with 167 member organisations, and the global trade union Public Services International (PSI).

The document calls for a “migrant-inclusive approach”, states that by including migrants, gaps in health and other inequities will be diminished and concludes that “The engagement of migrants themselves as key stakeholders in the community, will be a vital element for the sustainability of national plans.” This is followed by twelve “special considerations”, including non-discrimination and equitable access to health services and medical supplies including vaccines, gender-equality, age-sensitivity and equal treatment at the workplace. At the heart of the policy brief is a very comprehensive list of “recommended actions”—ten in total, with numerous subitems. These include very specific policy measures, many of them mirroring issues that had been brought up during the listening sessions—it can thus be said that they have shaped the output directed towards wider political spaces, including those which are more state-centric and thus less open for civil society participation. These policy measures include: “Identify hospitals to receive COVID-19 patients regardless of their migration status and prepare to mobilize surge acute and Intensive Care Unit (ICU) capacity”(3.1.b); “Put safeguards in place to ensure non-discriminatory, non-stigmatized border health screening at points of entry” (3.1.f); “dispel fears and misperceptions among local populations regarding migrants and COVID-19 outbreaks” (3.3.a); “Provide inclusive remote learning strategies for migrant children and their parents/caregivers” (3.5.a); “Develop capacities to map vulnerable migrants and their access to nutritious food” 3.8.d); “Ensure remittance services are labelled as essential services and that fees are reduced” 3.9.d) and “Protect workers in the workplace, irrespective of migration status, by strengthening occupational safety and health measures” (3.10.b).

## New stakeholders, central advocacy and policy issues

In sum, the listening sessions of the Network can be considered “invited spaces”. However, while they took place exclusively online, they have reached beyond what Kersting has called “demonstrative democracy”. They were on principal open to all stakeholders, although the usual *caveats* apply: interested participants would need a sufficient internet connection, possess the knowledge to navigate the registration process—and the awareness that those fora existed in the first place. It has to be acknowledged that the network strived for inclusiveness: translation was provided to a degree, different time zones taken into account and there was obvious diversity beyond the “usual suspects” (i.e. Geneva- or New York-based NGOS): participants had a fairly diverse profile with regard to geographic backgrounds and positions (i.e. support organisations as well as grassroots organizers). This observation was shared by Maulik: “it was important for me to see that new sectors of migrants came into the global migration space that had not really been included before”. Hence, the pandemic seemed to have opened up the invited spaces not only for a wider range of topics but also for aa broader spectrum of stakeholders.

This refers foremost to farmworkers, who obviously represent a significant population and play an important role in advocacy movements on labour or rights of indigenous people. In the specific migrant civil society space, though, farmworkers had so far been rarely discussed or represented. This could be attributed to the fact that a lot of migrant farmworkers, in Europe and elsewhere, fall under the category of seasonal workers, are rarely organized and generally fly under the radar of public attention. In the context of the pandemic the very same workers were suddenly considered to be “essential workers”. For example, Germany very much depends on such labour in the asparagus season which roughly falls into April and May—and thus coincided with the height of lockdown measures.^8^ As part of its EU Presidency, the country stressed the importance of protecting seasonal workers in the EU from exploitation, stating that those were not “second-class employees” and announced a strong stand against occupational health and safety abuses.^9^ In sum, governments and migrant civil society alike recognized the relevance of this sector and it remains to be seen how this realization will feature in longer-term policymaking and activism.

Regarding central topics that emerged during the consultations, access to services and firewalls were somewhat to be expected to be high on the agenda since they are very closely connected to health issues. However, the main topic that came up time and time again was regularization. According to one observer it was surprising how it could be talked about so openly and was taken seriously by all actors involved. This could be attributed to the fact that calls for increased regularization measures were not just mere advocacy points of migrant civil society but rooted in actual practices that were implemented at that time in several states. Especially the representatives of PICUM shared in several meetings examples of countries that had called for or already initiated (temporary) regularisation programs for undocumented workers such as Italy and Portugal or prevented people from becoming undocumented by extending residence permits during the lockdown such as Ireland, Greece, France and Poland. The country that received the most praise overall was Portugal which granted people with a pending residence application a temporary residence permit and thus access to the same services as Portuguese citizens, including unemployment benefits. PICUM compiled an overview of these various measures^10^ and called for more long-term regularisation programs, pointing out that regularisation might be “a taboo that isn’t one” since 24 out of 27 EU member states had implemented such programs in the past.^11^

Several observers shared the impression that the pandemic had “opened up the door” for a debate about regularization since the public perception of labour migrants shifted: “we started to see these people differently and their benefits to society in times of crises; that they are essential workers putting themselves at risk”. One observer pointed out that it would be now up to the policy advocates and stakeholders to move that issue forward with the goal of making some of these temporary changes more permanent and that the upcoming regional reviews for the GCM might be a good place to bring this argument to the floor. This demonstrates how the pandemic has opened the door for deliberations on this issue in two regards: first, directly by making the political issue more urgent. And, second, indirectly, by leading to the creation of new spaces for intervention such as the listening sessions or the “Voices from the Ground” platform.

While a more detailed mapping is beyond the scope of this paper, I have sketched out how several central issues that had been brought up during the sessions fed directly into the policy briefs issued by the Network and its workings groups thus underlining the strengthened input in global migration governance. The Network further issued on July 8 a statement “Standing in Solidarity with Migrants: Supporting Civil Society & other stakeholders in Responding to the COVID-19 Pandemic”.^12^ Here, the Network “salutes all actors providing vital protection, monitoring, advocacy, information and support to and in collaboration with migrants during the COVID-19 pandemic”. It notes that the exclusion of migrants leads to gaps in service provisions and that civil society and other stakeholders have stepped into the breach. What follows is a to-the point overview of the many forms in which migrant civil society has contributed to governance from below in times of the pandemic; examples include “providing multi-lingual information on COVID-19 adapted to the context migrants are living and working in, hotlines on gender-based violence and harassment, legal services and advice on complaint mechanisms, human rights monitoring, mental health support, training, advocacy and campaign support” (ibid.) Furthermore, stakeholders have “created solidarity networks and provide support to migrants, including food, water, essential medicine, shelter, personal protective equipment and economic assistance. They have established relief funds for farm workers, domestic workers and others who lost their livelihoods as a result of the pandemic.” (ibid.) In sum, migrant civil society is praised for providing critical assistance and a safety net in times when state measures were lacking, while at the same time being itself affected by the crises.

This is high praise indeed and shows that besides being able to influence the agenda, migrant civil society gained a lot of recognition at least in this *invited space*. The space provided by the Network thus expands beyond what is usually attributed to *invited spaces* in terms of openness and opportunities for engagement. The participants made use of these opportunities by bringing issues that were hitherto deemed controversial, most of all regularization, into the mainstream discourse. For example, while IOM had occasionally supported regularisation programs in the past, among others in Chile and Ecuador, before the pandemic the issue did not feature high on its agenda. In July 2020, though, the IOM African Capacity Building Centre (IOM ACBC) presented a webinar on “Migrant Regularization and other Measures to Address”. And in an October 2020 webinar hosted by IOM, on “Covid-19 and the transformation of migration and mobility globally”, researcher Luisa Feline Freier made a strong call for regularization: “From a public health perspective, regularization should cover all migrants and should not be limited in time.” [online presentation October 6, 2020; see also the commissioned paper (Freier 2020)].

The openness of these COVID-specific invited spaces notwithstanding, the question remains whether these may prove the exception or rather the rule in the longer run, especially when it comes to spaces where more concrete policy implementations are to be discussed, such as the review fora for the GCM.

## Invented spaces

If “invited spaces” are comparatively open as in the case of the “listening sessions” and provide potential to exert some influence on the agenda of more restricted- state-focused spaces—why is there a need to create “invented spaces” as well? One reason can be found in the way these were set up simultaneously to the invited spaces: not in an exclusive manner—on the contrary, there was noticeable overlap in participants and topics discussed. This is in line with the inside-outside strategy mentioned earlier; it could mean conducting deliberations outside of official fora in invented spaces and bringing the outcomes into invited spaces, thus strengthening the input into global migration governance; or vice versa, gathering resources in invited spaces and bringing them to the outside where they are shared with the wider constituency in invented spaces. The blurring of boundaries between the two spaces through the “zoomification” of consultations and deliberations could have contributed to this strategy: If there is easier access to and exchange between these spaces and issues and ideas can move more freely, it can be beneficial to set up a space where civil society exclusively controls the agenda.

An example for the first part of the strategy was the issuance of statements that were also shared in the invited spaces, including the “Voices from the Ground” website. On April 7, the Action Committee issued “A Global Civil Society Statement” under the heading “First, Save Lives: Solutions for the COVID-19 Pandemic and New Solidarity with Migrants and Refugees”.^13^ On behalf of its member networks, the preamble states “As leaders and organizations of civil society around the world—many of us ourselves migrants and refugees or their children and grandchildren—we urgently call on States and government authorities at all levels to protect migrants and refugees in this crisis.”

The statement then offers five larger areas of “cooperation and solutions”. The first of those refers to the title of the statement which is actually borrowed from the Migrants in Countries in Crisis (MICIC) Guidelines.^14^ These are a set of non-binding, voluntary principles, guidelines and effective practices for states and other stakeholders developed in 2015 and 2016 through a series of consultations and considered a good example for civil society participation in global governance processes.^15^ In making this connection, the civil society actors build upon existing documents, stating that the “First, save lives” “principle is fundamental and cross-cutting, drawn from international law, and should apply as the cornerstone for all COVID-19 pandemic responses related to migrants”. It is pointed out that the Emergency Response guidelines^16^ of the MICIC initiative provide step-by-step guidance and examples for multi-stakeholder actions in such crises.

Little is known whether states actually referred to these principles in the time of the pandemic, which highlights a major dilemma of global migration governance as well as a main advocacy area for migrant civil society: While the GCM and the MICIC were initially stated-led initiatives that produced tangible outcomes, it seems to be up to civil society to promote these, keep them on the agenda, urge states to refer to them. Many migrant civil society organisers started their advocacy work around the above-mentioned UN migrant workers convention and at some point they were the only ones left upholding the document and the need to ratify and implement it. Respondents were well aware that this can have a counter-productive effect, in governments having the perception that these are essential “civil society documents”, even if it was states in the first place who had negotiated, ratified, adopted or otherwise agreed to those. Stressing the importance of these principles and documents in “invented spaces” and linking them to “invited ones” is one way of reminding states of their commitments.

Not quite surprisingly, the need to “empower and partner directly with migrant and refugee communities and other civil society leaders” is also part of the over-arching principles of the action committee statements. Further aspects are 2. “essential health care and other protections”, including explicit reference to “firewalls”, 3. “Prevention and Other Effective Community Health Strategies” including the call to release migrants detained for immigration-related reasons and reaffirming the principle that “Children should never be sustained”, 4. “Economic and Social Solutions”, putting strong emphasis on labor rights and 5. and finally, calls for “a new solidarity”. In the spirit of the latter, the action committee stresses its openness to work with states, local authorities and other stakeholders. An addendum further compiles a “selection of good practices by states and inter-governmental bodies”, which is quite comprehensive considering the publication date of the statement; more of those are collected on a dedicated website.^17^ As Colin Rajah, Coordinator of the Action Committee, explains the rationale behind this addendum: “We really felt we should highlight good practices of member states and about 80 percent of that was on regularization, sometimes temporary, sometimes permanent, of migrant workers” (Interview Colin Rajah October 10, 2020).

It is obvious that there are several overlaps between the themes raised in this early statement and the outcomes of the deliberations in the invited spaces. This can be attributed to the interconnectedness of these spaces and quite fundamentally to the assessment that these are indeed the pressing issues and potential solutions on the ground. There is somewhat stronger wording in the civil society statement regarding detention and firewalls are explicitly mentioned. Significant conversion can be found on the issue of regularisation. According to Colin Rajah, Coordinator of the Action Committee, civil society had very much been advocating for this policy on the ground, along with access to basic services of health: “We cannot depend just on governments to do this on their own, we need to be able to support them, to push them, to provide them with statistics and facts and numbers to show them that this is should be beneficial for the economy, beneficial for the society at large not just the migrants”.

The Action Committee also organized several web meetings, starting on May 10 2020 with a “Webinar on COVID-19 and the Rights of Migrants”, attended by 277 participants. Alternatives to detention and a stocktaking of regularization measures were among the main points on the agenda with the explicit mention of PICUM bringing these points into the upcoming UN Network online session on the topic. These Network events were overall seen as a good opportunity, while activists were conscious of the fact that those were in the end still invited spaces. As one organizer put it, this means that in the end the invited spaces were still embedded in the UN system and the organizers had control over the proceedings, who was invited as input speaker etc. The organizer contrasted this with the civil society consultations on the GCM which were financially supported by member states through IOM, however, the organizing and participation modalities of the events had been completely left to the regional civil society networks to undertake. The organizers of these Regional Civil Society Consultations (RCSCs) were now concerned that the upcoming regional reviews of the GCM, which would also provide space for addressing the immediate and longer-term effects of the pandemic, might not follow the same standard. This was in part due to the decision to mandate the Regional Economic Commissions (RECs) of the UN with organizing the consultations—entities that were so far largely unknown spaces for migrant civil society and had limited experience in engaging with it.

The organizers of the 6 RCSCs therefore wrote an “Open Letter on Civil Society and Regional Migration Review Forums of the Global Compact for Migration” (2020, undated). Herein, they state that it “is essential that regional reviews continue to provide the space for meaningful, autonomous and robust civil society engagement”. The RECs should therefore ensure to consult civil society during the conception, design, and implementation of regional reviews. Furthermore, the participation of civil society in the review and its role in it should be determined by civil society itself and not by the UN. The signatories also refer to the move of most of the deliberations to online spaces due to the pandemic and ask that online reviews must allow for the equal participation of civil society actors: “We call for civil society to be directly involved in the actual review itself, not in separate or parallel reviews on the margins that may be organized or facilitated by the UN, and that civil society’s participation in the main review is done on an equal footing with governments and UN agencies. This includes guaranteeing an interactive environment where civil society is able to participate fully in panels and from the floor.” The initiative did seem to indeed have an effect on some of the regional consultations, as will be discussed in the concluding section. It also highlights civil societies efforts to influence the format of invited spaces.

Besides the Networks listening sessions, the Action committee continued to organise around other fora as well, such as through the classic (albeit online) format of holding a “Side Event to the High-Level Political Forum. Migration Indicators in the SDGs: COVID-19’s Impact on progress, and what is being done by various stakeholders” (July 17, 2020). During this event, according to Colin Rajah “there was a sense we need to go deeper into the objectives of the GCM, touch upon their interconnectedness, bring in expertise, the best speakers from member states, UN agencies, practitioners, civil society, academia to not just talk about policy in political context but also experiences on the ground.” The result was an ambitious webinar series that, over the course of 23 weeks, would “provide a multi-stakeholder space to review progress made on the implementation on each of the GCM objectives”.^18^ The program is organized by the Action Committee in cooperation with Migrant Forum in Asia (MFA), the Cross Regional Center for Refugees and Migrants (CCRM) and the Global Research Forum on Diaspora and Transnationalism (GRFDT). Besides being open to all interested parties, the webinars, streamed live and available for later viewings on youtube, are also at the core of a six-month Online Certificate Programme that is eligible to issue university credits. In terms of the comprehensiveness of disseminating information and analysing each of the 23 GCM goals this is a program unmatched by states or international institutions. These efforts could be seen as a case where the “invented spaces” become “reverse invited spaces”, i.e. where civil society sets the rules and brings representatives of governments, international organisations etc. to their “own turf”, presenting their agenda and hoping to win them as new allies.

Besides being the central coordinator for some campaigns, the Action Committee also picks up and promotes initiatives by its members. The main one with relation to COVID-19 was developed within an invented space and tackled the issue of wage theft. Spearheaded by the Action Committee member MFA, a large coalition of civil society organisations and trade unions launched an appeal on June 1, 2020, to establish an “Urgent Justice Mechanism” for migrant workers whose wages have been unjustly withheld by their employers. While this has been a perennial issue, the organizers of the campaign stress that the “COVID-19 pandemic has exacerbated the scale of wage theft against migrant workers in the context of cancelled contracts, high unemployment and forced repatriation”.^19^ The campaign started a website that does not only provide information but also offers the possibility to submit violations online.^20^ It has produced several appeals and policy documents on the issue, some of which were shared in the invited space of the listening sessions and the voices from the ground online platform.

## New stakeholders, central advocacy and policy issues

It has been shown that there has been significant overlap of the central policy issues between invited and invented spaces. To a degree, this can be expected—migrant civil society representatives have been present in both spaces, after all, and contributed to exchange between them, which led to mutually reinforcing the importance of issues such as regularization, access to services and alternatives to detention. It also shows how ideas and issues can travel between those spaces and how the “zoomification” has affected the boundaries between them. Thus, civil society pushed for the explicit mention of the concept of “firewalls”. With the campaign against wage theft, migrant civil society put an issue on the agenda that had not gained much attention in the other spaces; it was also an effort to link topics that had become particularly pressing during the pandemic to more fundamental issues. Hence, it was stated that wage theft is a long-standing issue that should be addressed beyond the corona crises mode.

Civil society can play a number of roles in global governance—including providing resources, acting as watchdog or transmission belt. Regarding the watchdog function, migrant civil society organisations continued to highlight human rights violations and humanitarian crises beyond the corona pandemic; for example, the Action Committee released a statement that called for an immediate humanitarian and human rights response to the Moria camp tragedy and presented it during the 45^th^ Human Rights Council on September 20, 2020.^21^ The need to “remind” member states of previous commitments and established tools such as the MICIC initiative, can also be considered part of the watchdog function. The role of resource providers can be linked to the transmission belt function—knowledge and advocacy goals from the grassroots level and member organisations of migrant civil society networks are brought into global fora, while, vice versa, the member organisations and migrants themselves are informed about these global processes. Besides hosting a number of webinars and distributing policy briefs, statements etc., the GCM objective review series can be considered a prime example of the transmission belt function, not the least since these online events bring together a varied number of stakeholders and open the space for direct online exchange.

## Conclusion

The pandemic has impacted the life of people all over the planet, reinforced and increased global inequalities and humanitarian crises in many fields, migration being one of them. It has also had an impact on how international and global policy deliberations are conducted. This paper has looked into the specific field of global migration governance. It has to be kept in mind that this is still an emerging field where obstacles such as the resentment of states towards perceived or actual infringements of their sovereignty are even more pronounced than in other areas of global governance. There is (with the possible exception of the subfield of refugees) no stable regime and no widely ratified binding human rights instrument. When migration slowly started to become a topic in the global arena through processes such as the GFMD, space for civil society was quite limited and there was a reluctance to talk about issues such as human rights of migrants or the situation of irregular migrants. Judging from this background, significant progress has been made in the past year with the GCM not as an end- but rather as a starting point for increased global migration governance.

If one accepts that such frameworks, may they be legally or just “morally” binding, can over time establish standards and influence policies on the ground (something the core human rights conventions in other fields have achieved), it does matter which issues are on the agenda and which are not. This is an area where migrant civil society has played a crucial role in the past two decades—by mainstreaming their agenda, influencing global discourses and documents such as the GCM. This might pale in comparison to the actual support migrant civil society actors provide on the ground; indeed, the critique that global migration governance mostly produces a stack of papers, statements and reports can be heard from activists as well as other stakeholders. This is a fair and unfair point at the same time: While what ultimately matters is the implementation on the ground, agreements on fundamental principles can also serve as an advocacy tool for migrant civil society. More importantly, the analysis of the core issues debated during the pandemic has shown that these were linked to very specific policy measures, good practices already established on the national level and references to existing documents and guidelines that could have led to better policies if they had been consulted.

“Could” is the crucial word here, though, since the GCM played a minor role at best during the first phase of the pandemic. As Williams Gois, regional coordinator of the MFA, put it during a webinar of its network referring to the situation in Asia: ”the GCM was meant to be a living document—it is now more dead than alive in dealing with the pandemic and needs to be resuscitated" (MFA Webinar on ““Returns, Repatriation, and Reintegration in the time of COVID-19”, May 14, 2020). This has not resulted in migrant civil society giving up on the GCM, though—quite the contrary: the GCM objective review webinar series is an impressive undertaking in raising knowledge and awareness of the compact. The open letter of the civil society organisers and continued advocacy in invited and invented spaces also had some effect on the inclusion of civil society in the regional reviews of the GCM. The first such review by the United Nations Economic Commission for Europe (UNECE) on November 12 and 13, 2020 was preceded by an “Informal Multi-stakeholder Consultation Regional Review of the GCM in the UNECE region” (November 8). The outcomes of these deliberations which match most of the central policy issues discussed in this paper and several references to the challenges posed by COVID-19, were presented by a PICUM representative to the participants of the actual review^22^ (which included all stakeholders—member states, UN agencies and civil society).

How did the “zoomification” of deliberations contribute to the process? For once, it was an enabling tool—the comparatively very open and inclusive listening sessions would not have been able to gather such a diverse group of stakeholders on a regular basis. Civil Society also used this tool for creating its own spaces—as Colin Rajah put it: ““We have been diversifying the time zones, so to speak” with no need for stakeholders to physically travel to Geneva or New York. While observers voiced concerns over the scope of inclusiveness and the issue of control in invited spaces, Kerstings observation of “demonstrative democracy” does not seem to apply to this particular field of online deliberations: The only instances of grandstanding and poisoned discourses I had to observe in over 40 meetings took place on the second day of the UNECE regional reviews, where the government representatives of Azerbaijan, Armenia and Turkey (ab)used the session for hostile statements on the Nagorno Karabakh conflict.

It goes without saying that the ultimate litmus test for global migration governance is its ability to improve the situation of people on the ground. I argue that during the—long—way of getting there, discourse matters too. Simply put, if a policy issue is not even mentioned on the international agenda, a potential solution is even more unlikely. On the repercussions of COVID-19 this paper has analysed a number of central issues and practical policies that were brought up in invited as well as in invented spaces..^23^

It has also shown how these issues and ideas can “travel” between invented and invited spaces. Distinguishing between these spaces and comparing them with regards to format, stakeholders and content provides us insights on how issues can emerge on the global agenda, on the strategies of migrant civil society and on ways to democratize global migration governance. It has been shown that civil society needs its own, independent spaces—having them “sponsored” through global institutions is no contradiction as long as there is no influence on the agenda and on the selection of participants. The knowledge on pressing issues, good practices and policy recommendations generated in these spaces can also be seen as a meaningful contribution to state-led processes, including “restricted spaces” where there is only limited civil society participation. While there is no substitute for in-person deliberations, it is likely that online deliberations will continue even when pandemic-related restrictions are lifted. They can be part of a hybrid approach to deliberations and enable continuous input from stakeholders between major in-person gatherings.

Finally, how can we at this stage assess the impact of COVID-19 on global migration governance? As I have argued in the beginning, the pandemic has clearly resulted in strengthening migrant civil societies’ role in the input dimension of global migration governance. We should not regard the pandemic as an isolated factor, though, but rather in interplay with existing trends and structures, acting in some cases almost as a catalysator. For example, it was certainly significant that the pandemic struck at a time when the newly formed UN migration network was still in the process of finding its feet, developing processes for interaction etc. While we can assume that the format for consultations with migrant civil society would have been further developed in any case, and some of these interactions might have been conducted online, the pandemic clearly has sped up this process, brought urgency and a new dynamic into it. Invited spaces were being opened up through “zoomification”, meetings were held more frequently, and civil society input has demonstrably shaped the outputs produced by the UN network and its members.

It would be hard to reverse this process after a perceived end of the pandemic, since COVID-19 has mostly intensified existing challenges in the policy field, and these will obviously remain pressing issues. The intensification of interactions can also be seen as a good practice in itself and as a trust-building measure between migrant civil society and the UN network.

Likewise, migrant civil society has held online meetings long before the pandemic, but the scope and outreach of its “invented spaces” has clearly grown as a result of COVID-19. The spaces can be seen as a pre-stage in the input dimension of global migration governance, where civil society gathers under its own rules and consolidates its agenda. They have a function besides preparing input for “invited spaces”, as has been shown by independently organized major campaigns such as the one against wage theft. I have also proposed the concept of “reverse invited spaces” which are invented by migrant civil society and to which representatives of governments, global institutions etc. are invited, presented with the agenda and possibly recruited as future allies.

On the content side of global migration governance, the pandemic has made existing problems worse—and thereby raised awareness of these issues. Topics formerly considered to be “taboos” such as regularization and access to services independent of status have entered the agenda of global migration governance, and in some cases travelled from invented to invited spaces of deliberations. This has also led to some changes in policies on the ground, but most of them were not a result of the output dimension of global migration governance but rather measures taken on the national and local level (quite often in cooperation with civil society). It was mostly migrant civil society—rather than states—that has fed these examples back into the global migration governance deliberations as “good practices”. Many of these such as regularization were temporary in nature, though, but the pandemic might have helped to establish them as a legitimate and appropriate tool. The coming years and the 2022 International Migration Review Forum in particular will show if the pandemic has had a lasting—and in this case potentially positive—effect on the output dimension of global migration governance.

## Data Availability

Data will not be shared – interviews fall partly or completely under Chatham House rules.

## Abbreviations

“Action Committee”: Civil Society Action Committee
CCRM: Cross Regional Center for Refugees and Migrants
CoM NGO: Committee on Migration
COVID-19: Coronavirus disease 2019
ECOSOC: United Nations Economic and Social Council
EU: European Union
GCM: Global Compact for safe, orderly and regular migration
GFMD: Global Forum on Migration and Development
GRFDT: Global Research Forum on Diaspora and Transnationalism
HLM UN: High-Level Meeting
ICMC: International Catholic Commission on Migration
ICRMW: International Convention on the Protection of the Rights of All Migrant Workers and Members of Their Families
ICU: Intensive Care Unit
IOM: International Organisation for Migration
IOM ACBC: IOM African Capacity Building Centre
MFA: Migrant Forum in Asia
MICIC: Migrants in Countries in Crisis
“The Network”: United Nations Network on Migration
NGO: Non-Governmental Organisation
PGA: People’s Global Action on Migration, Development and Human Rights
RCSC: Regional Civil Society Consultations
REC: Regional Economic Commissions
PICUM: Platform for International Cooperation on Undocumented Migrants
PSI: Public Services International
UAE: United Arab Emirates
UN: United Nations
UN-HLD: UN High-Level Dialogue on Migration and Development
WSFM: World Social Forum on Migration

## Footnotes

1 „When a meeting, or part thereof, is held under the Chatham House Rule, participants are free to use the information received, but neither the identity nor the affiliation of the speaker(s), nor that of any other participant, may be revealed.” https://www.chathamhouse.org/about-us/chatham-house-rule.

2 This term has gained some popularity, see for example https://verfassungsblog.de/toto-we-are-not-in-kansas-anymore/ Zoom has become a brand-turned-household name; although most meetings attended during the research for this article have been conducted on different platforms such as Webex or Bluejeans for data security concerns; UN meetings are often held on KUDO, a multilingual web conferencing platform that allows for simultaneous interpretation in the main languages.

3 https://migrationnetwork.un.org/about#.

4 Actually, two sessions were held to allow for participants from different time zones to participate in at least one of them; this approach was repeated in many of the following sessions.

5 https://migrationnetwork.un.org/statements/covid-19-does-not-discriminate-nor-should-our-response.

6 https://migrationnetwork.un.org/community-practice-voices-ground#.

7 https://www.migrationnetwork.un.org/sites/default/files/docs/final_network_wg_policy_brief_covid-19_and_access_to_services_1.pdf.

8 Plans for locals to fill their place were quickly scrapped amidst the realization that this was actually very skilled labour if one wanted to avoid damaging the crops. As a result, Germany organized special flights for up to 80 000 seasonal workers from eastern Europe to come into the country https://www.dw.com/en/germany-flies-in-seasonal-workers-to-harvest-asparagus/av-53249738.

9 https://www.bmas.de/EN/EU-Presidency/SeasonalWorkers/seasonal-workers.html.

10 https://picum.org/wp-content/uploads/2020/10/Non-exhaustive-overview-of-European-government-measures-impacting-undocumented-migrants-taken-in-the-context-of-COVID-19.pdf.

11 https://picum.org/regularising-undocumented-people-in-response-to-the-covid-19-pandemic/.

12 https://migrationnetwork.un.org/standing-solidarity-migrants-supporting-civil-society-other-stakeholders-responding-covid-19.

13 https://csactioncommittee.org/wp-content/uploads/2020/04/Civil-Society-Statement-on-COVID-19-and-Migrants-final.pdf.

14 https://micicinitiative.iom.int/guidelines.

15 http://gcmigration.org/micic/.

16 https://micicinitiative.iom.int/sites/default/files/document/micic_guidelines_english_web_13_09_2016.pdf#page=34.

17 https://csactioncommittee.org/covid-19-good-practices/.

18 https://csactioncommittee.org/gcm-objectives-webinar-series/.

19 https://mfasia.org/appeal-5-measures-for-addressing-wage-theft-affecting-millions-of-migrant-workers-in-the-times-of-covid-19/.

20 https://justiceforwagetheft.org/.

21 https://csactioncommittee.org/greece-and-eu-must-take-action-now/.

22 https://csactioncommittee.org/wp-content/uploads/2020/11/GCM-UNECE-Regional-Review-Multistakeholder-meeting-Report-by-Rapporteur_Michele.pdf.

23 The need for such spaces and their discourses can be illustrated by a recent report (Papademetriou 2020) of the influential Migration Policy Institute: Hyped by the institution as “THE definitive piece on migration during and after COVID” the report stresses border controls, returns and the problem of “illegal immigration”—but lacks a single reference to the central issues discussed in this paper: regularisation, access to services, alternatives to detention, wage theft and even the GCM. https://twitter.com/meghan_benton/status/1328045924367691779.
