# Correction to: Global migration governance from below in times of COVID-19 and “Zoomification”: civil society in „invited “ and „invented “ spaces

**DOI:** 10.1186/s40878-022-00312-1

**Published:** 2022-11-10

**Authors:** Stefan Rother

**Affiliations:** University of Freiburg, Rempartstr. 15, 79085 Freiburg, Germany

## Correction to: Comparative Migration Studies (2022) 10:1 https://doi.org/10.1186/s40878-021-00275-9

In the original publication of this article the issue details of the references were not available. The article has been updated to correct this.

